# Differentials in death count records by databases in Brazil in 2010

**DOI:** 10.11606/s1518-8787.2022056004282

**Published:** 2022-10-19

**Authors:** Victor Hugo Dias Diógenes, Elzo Pereira Pinto, Marcos Roberto Gonzaga, Bernardo Lanza Queiroz, Everton E. C. Lima, Lilia Carolina C. da Costa, Aline S. Rocha, Andrêa J. F. Ferreira, Camila S. S. Teixeira, Flávia Jôse O Alves, Leila Rameh, Renzo Flores-Ortiz, Alastair Leyland, Ruth Dundas, Maurício L. Barreto, Maria Yury Travassos Ichihara

**Affiliations:** I Universidade Federal do Rio Grande do Norte Centro de Ciências Exatas e da Terra Programa de Pós-Graduação em Demografia Natal RN Brasil Universidade Federal do Rio Grande do Norte . Centro de Ciências Exatas e da Terra . Programa de Pós-Graduação em Demografia . Natal , RN , Brasil; II Universidade Federal da Paraíba Centro de Ciências Sociais Aplicadas Departamento de Finanças e Contabilidade João Pessoa PB Brasil Universidade Federal da Paraíba . Centro de Ciências Sociais Aplicadas . Departamento de Finanças e Contabilidade . João Pessoa , PB , Brasil; III Fundação Oswaldo Cruz Centro de Integração de Dados e Conhecimentos para Saúde Salvador BA Brasil Fundação Oswaldo Cruz . Centro de Integração de Dados e Conhecimentos para Saúde . Salvador , BA , Brasil; IV Universidade Federal do Rio Grande do Norte Centro de Ciências Exatas e da Terra Departamento de Demografia e Ciências Atuariais Natal RN Brasil Universidade Federal do Rio Grande do Norte . Centro de Ciências Exatas e da Terra . Departamento de Demografia e Ciências Atuariais . Natal , RN , Brasil; V Universidade Federal de Minas Gerais Faculdade de Ciências Econômicas Centro de Desenvolvimento e Planejamento Regional Belo Horizonte MG Brasil Universidade Federal de Minas Gerais . Faculdade de Ciências Econômicas . Centro de Desenvolvimento e Planejamento Regional . Belo Horizonte , MG , Brasil; VI Universidade Estadual de Campinas Instituto de Filosofia e Ciências Humanas Núcleo de Estudos de População Campinas SP Brasil Universidade Estadual de Campinas . Instituto de Filosofia e Ciências Humanas e Núcleo de Estudos de População . Campinas , SP , Brasil; VII Universidade Federal da Bahia Instituto de Matemática e Estatística Departamento de Estatística Salvador BA Brasil Universidade Federal da Bahia . Instituto de Matemática e Estatística . Departamento de Estatística . Salvador , BA , Brasil; VIII Universidade Federal da Bahia Escola de Nutrição Programa de Pós-Graduação em Alimento, Nutrição e Saúde Salvador BA Brasil Universidade Federal da Bahia . Escola de Nutrição . Programa de Pós-Graduação em Alimento, Nutrição e Saúde . Salvador , BA , Brasil; IX Universidade Federal da Bahia Instituto de Saúde Coletiva Programa de Pós-Graduação em Saúde Coletiva Salvador BA Brasil Universidade Federal da Bahia . Instituto de Saúde Coletiva . Programa de Pós-Graduação em Saúde Coletiva . Salvador , BA , Brasil; X University of Glasgow Medical Research Council Glasgow Scotland University of Glasgow . Medical Research Council . Glasgow , Scotland

**Keywords:** Mortality Registries, Information Storage and Retrieval, Health Information Systems

## Abstract

**OBJECTIVE:**

To compare the death counts from three sources of information on mortality available in Brazil in 2010, the Mortality Information System (SIM - *Sistema de Informações sobre Mortalidade* ), Civil Registration Statistic System (RC - *Sistema de Estatísticas de Resgistro Civil* ), and the 2010 Demographic Census at various geographical levels, and to confirm the association between municipal socioeconomic characteristics and the source which showed the highest death count.

**METHODS:**

This is a descriptive and comparative study of raw data on deaths in the SIM, RC and 2010 Census databases, the latter held in Brazilian states and municipalities between August 2009 and July 2010. The percentage of municipalities was confirmed by the database showing the highest death count. The association between the source of the highest death count and socioeconomic indicators - the *Índice de Privação Brasileiro* (IBP – Brazilian Deprivation Index) and *Índice de Desenvolvimento Humano Municipal* (IHDM – Municipal Human Development Index) - was performed by bivariate choropleth and Moran Local Index of Spatial Association (LISA) cluster maps.

**RESULTS:**

Confirmed that the SIM is the database with the highest number of deaths counted for all Brazilian macroregions, except the North, in which the highest coverage was from the 2010 Census. Based on the indicators proposed, in general, the Census showed a higher coverage of deaths than the SIM and the RC in the most deprived (highest IBP values) and less developed municipalities (lowest IDHM values) in the country.

**CONCLUSION:**

The results highlight regional inequalities in how the databases chosen for this study cover death records, and the importance of maintaining the issue of mortality on the basic census questionnaire.

## INTRODUCTION

Mortality indicators are critical to inform and monitor government public policies, especially those related to health. However, we emphasize the importance of using reliable estimates which reflect the epidemiological and demographic profile of the geographic areas studied ^1 , 2^ . Therefore, a high-quality mortality information system which accurately registers all the occurring deaths is essential. Hence, an investigation into the quality of death statistics is important for researchers and demographers of population health ^3^ .

In Brazil, mortality studies that assess data from municipalities in the entire national territory may include three main information sources: the *Sistema de Informações sobre Mortalidade* (SIM – Mortality Information System) and the *Sistema de Estatísticas do Registro Civil* (RC – Civil Registration Statistic System), both compiled from continuous administrative death records ^4^ ; and the *Instituto Brasileiro de Geografia e Estatístic* a (IBGE – Brazilian Institute of Geography and Statistics) 2010 Demographic Census, which includes information on deaths during the reference period of the census research ^5^ and considers the enumeration of death counts, unlike both SIM and RC.

However, one of the main problems related to the quality of information on deaths in Brazil is the under-recording of the event ^6 , 7^ , which may compromise estimates of mortality statistics, resulting in inaccurate indicators. Although this problem has decreased considerably in recent decades ^8 , 9^ , significant regional differences remain in the coverage of information ^10 , 11^ , which may relate to socioeconomic circumstances ^12^ . Moreover, there are several reasons for under-recording, and these may vary according to the database used ^4^ .

Historically, the comparison between the SIM and RC databases is used to validate and confirm the development and consolidation of both administrative registration systems ^4 , 9 , 13 , 14^ . However, only a limited number of studies compare these administrative death records (SIM and RC) with 2010 Census data. Some research has evaluated the quality of census mortality data ^12^ , or used this source to verify mortality differentials in the population and compare them with SIM data ^15^ . These databases use distinct death collection methods and they are subject to different under-notification errors, comparing the administrative registration systems and the 2010 Demographic Census may help us to understand the extent of under-registration across these different systems. Thus, we would be able to determine and quantify associations between specific characteristics of the areal unit of analysis and notification.

This study aims to conduct a comparative analysis of the death count in the SIM, RC, and 2010 Census databases by region, state, and municipality, and to assess the spatial association between the number of deaths recorded on each database and the socioeconomic characteristics of the municipalities in relation to the source of information with the highest death count. Our specific research questions are: Which database gathers more information on deaths in Brazil? Are there any differences in the absolute number of deaths if we consider the various subnational geographic levels? How different are the database coverages based on socioeconomic strata?

## METHODS

This study conducted a descriptive and comparative analysis of the number of deaths registered by the SIM, RC, and enumerated in 2010 Census database, the latter held during the reference period of the census research (August 2009 to June 2010) at different geographical levels (nation, regions, states, and municipalities). The 2010 Census gathered information on deaths which had taken place in households in the last 12 months, including the age and sex of the person who had passed away. The inclusion of this information was listed in the suggestions put forward by the United Nations in 1997, in the document “Principles and Recommendations for Population and Housing Censuses” for the round of 2000 and 2010 censuses ^16^ . The primary source for the SIM and RC data is the declaration of death, a document usually issued by a health institution. SIM data were downloaded from the public DATASUS website (https://datasus.saude.gov.br/mortalidade-desde-1996-pela-cid-10), and RC and 2010 Census data, from the IBGE website (https://sidra.ibge.gov.br/home/pnadcm).

The spatial association between the socioeconomic characteristics of municipalities and the highest death count of a database was estimated using two geostatistical methods. The first, descriptive, method comprised the construction of bivariate choropleth maps which use a matrix of colours to simultaneously represent, on a continuous scale, the values of two variables in a geographical space.

The second method was based on local bivariate spatial dependence, in which the value of a variable in one region is correlated in space with the values of neighbouring regions. The Moran Bivariate Local Index of Spatial Association - LISA - was used for this purpose ^17^ , from which the bivariate cluster maps derive. Spatial clusters can be identified by these maps, in which a statistically significant association between the values of a variable of a particular municipality and those of a neighbouring one can be observed.

In total, two socioeconomic measures available for municipalities were used. The *Índice de Privação Brasileiro* (IBP – Brazilian Deprivation Index) and the *Índice de Desenvolvimento Humano Municipal* (IHDM – Municipal Human Development Index). The IBP is a measure of material deprivation comprising income, educational attainment, and household conditions ^18^ . The IDHM, an indicator prepared by the United Nations Development Programme (UNDP), is a composite measure of human development in municipalities comprising the population’s lifespan, income, and education.

These indicators were spatially associated with the ratio of deaths recorded by the SIM and the Census (SIM/Census), considering that these databases showed the largest collection of information on deaths in most analyzed areas. This ratio indicates the extent to which SIM records vary from the Census.

Information on deaths is collected in municipalities, so the number of deaths in the states (Distrito Federal - DF), regions, and the whole country is obtained by the sum of these records for their respective municipalities. Therefore, there are slight differences in the number of SIM and RC registrations if data are directly collected by total amount for the DF, region, or Brazil, since they registered deaths in which the municipality was not identified. These differences failed to affect the analyses or conclusions of this study. We should clarify that our research refers to the death count from one source in relation to another, and not to the overall coverage in relation to the true number of deaths, which is unknown.

Microsoft Excel, R, and GeoDa were used for data analysis, and QGis and R to produce the data spatialization maps.

This study exclusively used secondary data in the public domain and, therefore, approval by a Research Ethics Committee was unrequired.

## RESULTS

The SIM database shows a higher total death count in Brazil (1,114,568) than the other information sources ( Table 1 ). The proportion of deaths registered in the SIM was 7.75% higher than the Census (1,034,418) and 1.8% higher than the RC (1,094,866). The SIM showed the highest death count in the Brazilian Midwest, Northeast, and Southeast. The RC registered the highest death count in the South, although the difference between this database and the SIM was slight. The Census showed a higher death count in the North than the SIM, which registered 91.12% of the deaths recorded by the census research ( Table 1 ).

**Table 1:** Deaths in Brazil and its macroregions between August 2009 and July 2010, according to the registration source.

Region	Census	RC	SIM	Database with the highest death count	SIM/Census (%)	SIM/RC (%)
Brazil	1,034,418	1,094,866	1,114,568	SIM	107.75	101.80
North	70,034	58,904	63,813	Census	91.12	108.33
Northeast	276,535	265,576	278,710	SIM	100.79	104.95
Southeast	456,358	521,768	523,114	SIM	114.63	100.26
Midwest	69,193	70,649	71,099	SIM	102.75	100.64
South	162,298	177,969	177,832	RC	109.57	99.92

SIM: *Sistema de Informações sobre Mortalidade;* RC: *Sistema de Estatísticas do Registro Civil; IBGE: Instituto Brasileiro de Geografia e Estatística.*

Source: The 2010 Demographic Census (IBGE), the Civil Registry (IBGE), and the SIM/Datasus/Ministry of Health (MS).

The SIM was the database with the highest death count in 14 states, the census in 8, and RC in 5 ( Table 2 ). Generally speaking, the states in which the SIM has the highest death count are in the Southeast, Northeast, Midwest, and South. The demographic census showed the highest number of deaths predominantly in states in the North, corroborating the results Table 1 shows.

**Table 2:** Deaths by state between August 2009 and July 2010, according to the registration source.

State	Region	Census	RC	SIM	Database with the highest death count	SIM/Census (%)	SIM/RC (%)
GO	Midwest	31,289	32,322	31,549	RC	100.83	97.61
MS	Midwest	13,901	13,631	14,172	SIM	101.95	103.97
MT	Midwest	14,038	14.16	14,697	SIM	104.69	103.79
DF	Midwest	9,965	10,536	10,681	SIM	107.19	101.38
AP	North	2,771	2.22	2,066	Census	74.56	93.06
PA	North	34.65	27,009	30,643	Census	88.44	113.45
AM	North	14.27	11,899	12,985	Census	91.00	109.13
TO	North	6,741	6,051	6,336	Census	93.99	104.71
RR	North	1,697	1,518	1,597	Census	94.11	105.20
RO	North	7,022	7,229	7,194	RC	102.45	99.52
AC	North	2,883	2,978	2,992	SIM	103.78	100.47
MA	Northeast	31,765	20,181	25,293	Census	79.63	125.33
BA	Northeast	75,459	73,401	74,198	Census	98.33	101.09
RN	Northeast	16,224	15,064	16,053	Census	98.95	106.57
PB	Northeast	21,685	23,222	22,716	RC	104.75	97.82
PI	Northeast	15,207	13,511	15,285	SIM	100.51	113.13
AL	Northeast	17,206	15,682	17,424	SIM	101.27	111.11
CE	Northeast	40.82	41,151	43.2	SIM	105.83	104.98
SE	Northeast	10,008	9,868	10.85	SIM	108.41	109.95
PE	Northeast	48,161	53,496	53,691	SIM	111.48	100.36
RS	South	68,608	77,853	77,238	RC	112.58	99.21
SC	South	32,835	34,406	34.49	SIM	105.04	100.24
PR	South	60,855	65.71	66,104	SIM	108.63	100.60
MG	Southeast	109,091	118,986	116,782	RC	107.05	98.15
ES	Southeast	18.4	20,433	20,755	SIM	112.80	101.58
SP	Southeast	223,009	258,955	260,941	SIM	117.01	100.77
RJ	Southeast	105,858	123,394	124,636	SIM	117.74	101.01
**Total**		**1,034,418**	**1,094,866**	**1,114,568**	**SIM**	**107.75**	**101.80**

SIM: *Sistema de Informações sobre Mortalidade* ; RC: *Sistema de Estatísticas do Registro Civil* ; IBGE *: Instituto Brasileiro de Geografia e Estatística.*

Source: The 2010 Demographic Census (IBGE), the Civil Registry (IBGE), and the SIM/Datasus/Ministry of Health (MS).

We found the greatest differences between the SIM and census records (SIM/Census ratio under 90%) in the states of Maranhão, Amapá, and Pará. Note that Amapá is the only state in which the SIM reports the lowest death count, representing only 74.56% and 93.06% of the deaths registered in the Census and the RC, respectively.

Analysis at the municipal level highlighted that most municipalities (42.52%) had the SIM as the database with the largest collection of information on deaths. Of the 449 municipalities in the North, 65.48% had the Census as the database with the highest relative coverage. In the Northeast and Midwest, the Census was the database with the highest number of deaths in most municipalities, at 44.43% and 39.27% respectively. However, the SIM showed the highest number of total deaths in these regions. This apparent contradiction is due to the different weights municipalities have in the total number of deaths. The same pattern takes place in the South, in which the database with the highest relative death record was the RC, although most municipalities (50.51%) had the SIM as the database which gathered more information on deaths. Lastly, in the Southeast, the SIM had the highest relative coverage for both the number of deaths and municipalities (50.3%).

**Figure 1:**
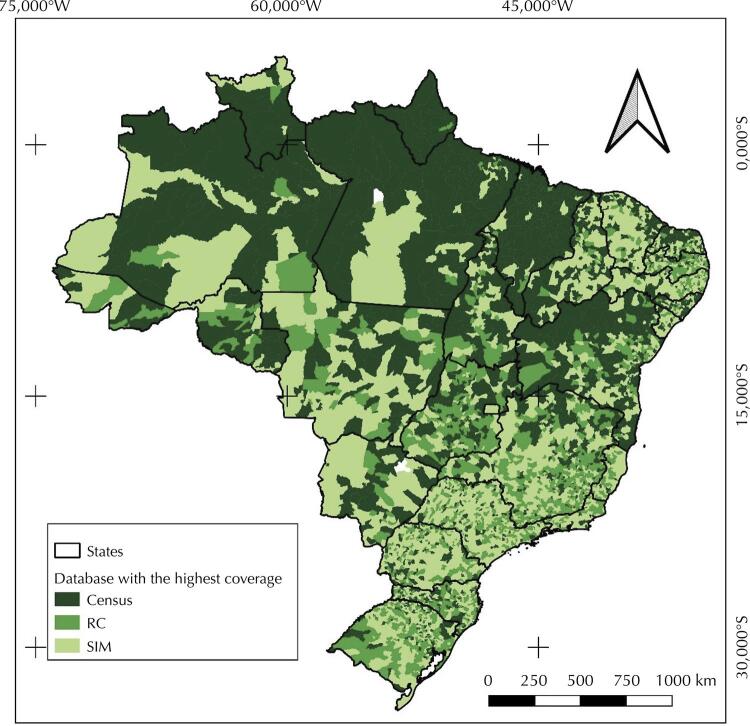
Brazilian municipalities according to the database with the highest death count, 2010.

IBGE: *Instituto Brasileiro de Geografia e Estatístic* a; SIM: *Sistema de Informações sobre Mortalidade* ; RC: *Sistema de Estatísticas do Registro Civil* .

Source: 2010 Demographic Census (IBGE), the Civil Registry (IBGE), and the SIM/Datasus/Ministry of Health (MS) data.

We visually find a concentration of the Census as the database with the highest death count in the municipalities of the North and in some areas of the Northeast (Maranhão, northern Rio Grande do Norte, and northern and western Bahia) ( Figure 1 ). The SIM prevails as the database with the highest death count in Southern and Southeastern municipalities.

**Figure 2:**
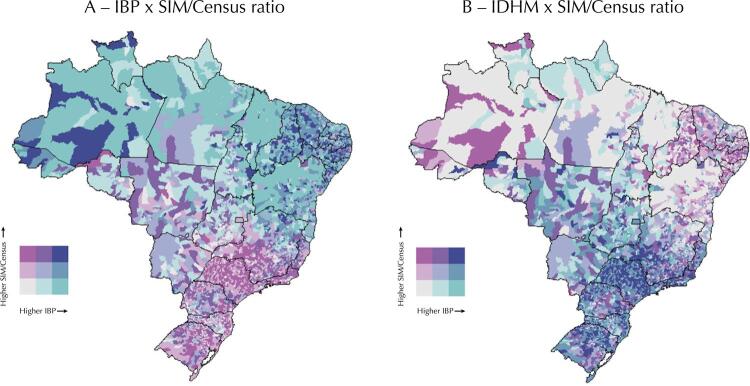
Socioeconomic indicators (IBP and IDHM) X SIM/Census ratio, Brazil, 2010.

IBGE: *Instituto Brasileiro de Geografia e Estatístic* a; SIM: *Sistema de Informações sobre Mortalidade;* IBP: *Índice de Privação Brasileiro;* IDHM *: Índice de Desenvolvimento Humano Municipal.*

Source: 2010 Demographic Census (IBGE), Civil Registry (IBGE), and SIM/Datasus/ Ministry of Health (MS) data.

In relation to the association between the predominance of the SIM or the Census and socioeconomic indicators, we found that, in general, the more materially deprived (highest IBP values) and less developed (lowest IDHM values) municipalities are in the Brazilian North and Northeast, and most show low SIM/Census ratio values, suggesting a higher Census coverage than the SIM ( Figure 2 ). However, the less deprived and more developed municipalities are in the South and Southeast whose SIM/Census ratio values are predominantly high.

These results suggest an association between the database with a higher death count and the level of socioeconomic development of Brazilian municipalities. The Census reported more deaths than the SIM in the more deprived and less developed municipalities, whereas the SIM reported more deaths in the municipalities with higher socioeconomic levels. Exceptions to this pattern are the municipalities located more to the west of the Brazilian North and the towns in the countryside of the states of Piauí, Ceará, Paraíba, and Pernambuco since they show high IBP and low IDHM values and have the SIM as the database with the highest coverage.

**Figure 3:**
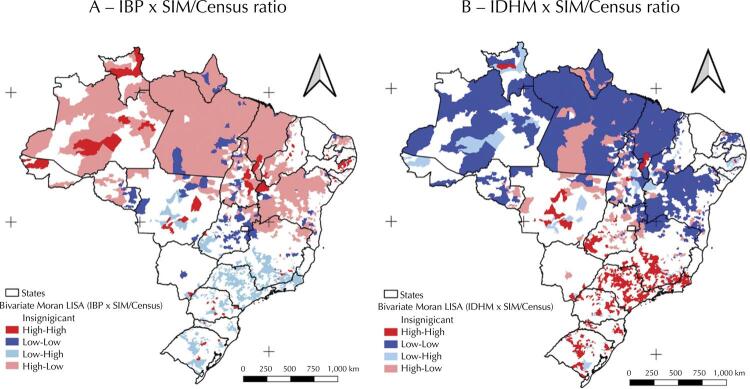
Socioeconomic indicator (IBP and IDHM) bivariate cluster and SIM/Census ratio map, Brazil, 2010.

IBGE: *Instituto Brasileiro de Geografia e Estatístic* a; SIM: *Sistema de Informações sobre Mortalidade;* IBP: *Índice de Privação Brasileiro;* IDHM *: Índice de Desenvolvimento Humano Municipal.*

Source: 2010 Demographic Census (IBGE), Civil Registry (IBGE), and SIM/Datasus/Ministry of Health (MS) data.

We find a similar result if we consider the Moran LISA bivariate cluster maps ( Figure 3 ). Considering the IBP ( Figure 3a ), we can confirm that the spatial clusters in pink consist of municipalities with high material deprivation values. Their neighbours show low SIM/Census ratio values (High-Low), and are mostly concentrated in the North and some areas of the Northeast. The areas in light blue, mainly located in the South and Southeast, consist of municipalities with a low IBP, and its neighbours show high SIM/Census values (Low-High). This method can also identify discrepancies. For example, the dark blue areas consist of municipalities with a low IBP, and their neighbours show low SIM/Census values (Low-Low).

If we consider the IDHM ( Figure 3b ), we note an overlap of the highlighted areas. The dark blue areas consist of municipalities with a low IHDM which are surrounded by municipalities with a lower SIM/Census ratio (Low-Low). The dark red areas, notably concentrated in the Southeast, are municipalities with a high IDHM which are surrounded by cities with higher SIM/Census ratio values.

These results indicate that the presence of the Census as the database with the highest coverage is spatially related to the low development indices of neighbouring municipalities, and these spatial clusters are mainly concentrated in the North and, to a lesser extent, the Northeast.

## DISCUSSION

Our analysis shows that the highest death count in the 2010 Demographic Census, compared to the SIM and RC, is concentrated in the Brazilian North and, to a lesser extent, the Northeast, observed in the less developed and more materially deprived municipalities. These results suggest that the database with the highest registration of deaths is associated with the socioeconomic characteristics of these municipalities, and that there is a clear pattern of spatial distribution in Brazil. We highlight that those socioeconomic levels and location are considerably correlated characteristics in the Brazilian territory, with the North and Northeast having the most deprived and less developed municipalities in the country. Therefore, the main finding of this study was that the lower the socioeconomic level of the municipality, the greater the possibility of the Census reporting a higher number of deaths, compared to the other databases. This fact may indicate that factors such as the municipal infrastructure in the health authorities (and others, such as the presence of a registry office) may severely impact the flow of SIM and RC registrations in relation to the Census.

Contrary to this overall trend, some of the municipalities in the Northeastern countryside, even with low socioeconomic levels, show a higher death count in the SIM than in the Census. Future studies should further explore this fact, but one hypothesis suggests this may be related to a programme to reduce the registration of deaths with poorly defined causes. This programme focused on the Brazilian North and Northeast and aimed to improve the collection and quality of information on deaths gathered by the SIM in these two regions, though it achieved more successful results in the Northeast ^19^ .

The literature reports failures in local mortality registration systems as one of the relevant reasons for the specific under-recording of deaths in the SIM ^3 , 4^ . There may also be cases in which families, even when they have the declaration of death, take time to issue (or even fail to acquire one) it at the Civil Registry Office, contributing to the under-recording in the RC database. Under-recording on both databases is associated with the existence of burials without the required documentation (such as death certificates) in irregular cemeteries, which still exist in poorer regions and rural areas ^20^ . These facts may partly explain why the Census reported more deaths in these regions than the SIM and the RC.

When comparing only the SIM with the RC, the former has a higher coverage in most territories, especially in the North and Northeast. Even with improvements and the convergence of RC and SIM coverage in recent decades ^3 , 4 , 9^ , there are still relevant regional disparities between them, mainly in favour of the SIM.

Despite official death registration systems, the inclusion of an item on death at home in the basic 2010 Census questionnaire provided a new possibility for mortality analyses ^21^ . We must highlight that the inclusion of this item was a suggestion by the United Nations for the round of subsequent censuses to try and obtain better mortality records for countries with lower-quality vital systems.

A major advantage of the Census is its capillarity, since, in theory, it covers the entire national territory. Thus, the items contained on the basic questionnaire enable a geographical breakdown of the data to its lowest levels. This characteristic offers a great opportunity for studies that aim to identify socioeconomic differences and inequities, particularly in health, in addition to research focused on mortality estimates in small areas ^25^ and mortality differentials by a range of population characteristics ^26 , 27^ .

In this context, the findings of this study suggest that the Census gained relevance, in relation to the SIM and the RC, in registering deaths in smaller and less developed municipalities, particularly those located in Northern Brazil, and also highlights the importance of the census research in regions with these sociodemographic characteristics. Furthermore, considering the recent discussion on the reduction of the number of items on census questionnaires, we highlight the immense value of retaining the mortality item on the basic questionnaire.

Despite this potential, census mortality data have limitations, such as its 10-year frequency; the 12-month reference period for deaths which fail to coincide with the year end of the calendar year; the under-recording of deaths caused by errors in understanding the reference period (since a household resident provides the information); the disregard of deaths, especially of new-born children; and the possibility of termination or dissolution of the household following the death of an individual ^12 , 22^ .

Moreover, as an inconsistency in the census data, we highlight that there may be errors when declaring the age of the deceased and of the people who live in the household ^28^ . Although this fails to influence the total death count, this problem may interfere in the age estimates of the mortality structure ^29^ .

We should clarify that census research should not be seen as a solution to this problem, but as an alternative to complement death data sources, and not lessen the importance of ensuring the existence of quality systems to record these crucial events. In this context, we can confirm that there is a consensus between mortality data users that the inclusion of the item recording a death in the household in the basic census questionnaire was of the utmost importance for studies on the topic, providing new possibilities for estimating mortality levels and structure which reflect a more likely reality.

In the context of the covid-19 pandemic, for example, the 2022 Brazilian demographic census data may provide an alternative to analyze its impact on mortality by estimating excess mortality and assist researchers in understanding the relation of the pandemic with other demographic variables.

As a research limitation, we highlight that the deaths gathered are included in the reference period for the last census, resulting in a scenario for mortality dating back more than 10 years. It is unlikely that there have been any significant changes in the structure and level of mortality in Brazil, especially at the highest geographical levels. However, differences in coverage between the SIM and the RC may be less accentuated than what this study ascertained, considering the continuous tendency of a convergence between the two databases observed in recent years.

As a recommendation for future studies that aim to estimate mortality in small areas, we suggest a conjugation of the Brazilian databases of death notification, considering that database which gathered the highest number of deaths by gender and age in each municipality. Although this would only be possible in years censuses are held, this approach may provide better and more accurate mortality measures.

We hope the results and the discussion of this study equip users of mortality data with important recommendations. We highlight that research should pay special attention to the selection and use of Brazilian mortality databases since the region studied and/or information database chosen may produce specific mortality outcomes. We must highlight that the quality of mortality records has evolved in recent years. However, we still observe a regional variability in data quality, and an evaluation of data quality based on the different methodologies available is, therefore, important.
